# Multiple Sclerosis Is Associated with Immunoglobulin Germline Gene Variation of Transitional B Cells

**DOI:** 10.32607/actanaturae.11794

**Published:** 2022

**Authors:** Y. A. Lomakin, L. A. Ovchinnikova, M. N. Zakharova, M. V. Ivanova, T. O. Simaniv, M. R. Kabilov, N. A. Bykova, V. S. Mukhina, A. N. Kaminskaya, A. E. Tupikin, M. Y. Zakharova, A. V. Favorov, S. N. Illarioshkin, A. A. Belogurov, A. G. Gabibov

**Affiliations:** Shemyakin-Ovchinnikov Institute of Bioorganic Chemistry RAS, Moscow, 117997 Russia; Research Center of Neurology, Moscow, 125367 Russia; Institute of Chemical Biology and Fundamental Medicine SB RAS, Novosibirsk, 630090 Russia; Vavilov Institute of General Genetics RAS, Moscow, 119991 Russia; Institute for information transmission problems RAS, Moscow, 127051 Russia; A.I. Yevdokimov Moscow State University of Medicine and Dentistry, Moscow, 127473 Russia; Lomonosov Moscow State University, Moscow, 119991 Russia

**Keywords:** multiple sclerosis, neurodegeneration, immunoglobulin, transitional B cells, NGS, regulatory B cells, BCR-Seq, germline, TrB

## Abstract

The regulatory functions of the B-cell compartment play an important role in the development and suppression of the immune response. Disruption of their anti-inflammatory functions may lead to the acceleration of immunopathological processes, and to autoimmune diseases, in particular. Unfortunately, the exact mechanism underlying the functioning and development of regulatory B cells (Breg) has not yet been fully elucidated. Almost nothing is known about their specificity and the structure of their B-cell receptors (BCRs). In this research, we analyzed the BCR repertoire of the transitional Breg (tBreg) subpopulation with the CD19^+^CD24^high^CD38^high^ phenotype in patients with multiple sclerosis (MS), using next-generation sequencing (NGS). We show, for the first time, that the immunoglobulin germline distribution in the tBreg subpopulation is different between MS patients and healthy donors. The registered variation was more significant in patients with a more severe form of the disease, highly active MS (HAMS), compared to those with benign MS (BMS). Our data suggest that during MS development, deviations in the immunoglobulin Breg repertoire occur already at the early stage of B-cell maturation, namely at the stage of tBregs: between immature B cells in the bone marrow and mature peripheral B cells.

## INTRODUCTION

Multiple sclerosis (MS) is one of the most common chronic autoimmune diseases of the central nervous system (CNS). It affects more than 2.3 million people worldwide [1]. Its triggering mechanism, and its mechanism of immune-mediated neurodegeneration in particular, still remains unknown, which causes significant difficulties in efforts to design a strategy for MS treatment and drug development [2 , 3, 4]. Since MS was discovered, the main role in its pathogenesis has been assigned exclusively to the T cell-mediated immunity. However, over the past decade, a lot of evidence has emerged confirming that B cells are directly involved in the development of autoimmune processes, including MS [5]. MS patients have been shown to have elevated titers of autoreactive antibodies that specifically recognize native components of the myelin sheath. Moreover, the catalytic immu noglobulins that hydrolyze the myelin basic protein (MBP), one of the characteristic autoantigens in MS, have also been found exceptionally in MS patients, as opposed to healthy donors or patients with other neurodegenerative diseases [6, 7, 8]. Despite the long history of MS research, its exact etiology still remains elusive. Molecular mimicry, epitope spreading, and cross-reactivity are believed to underlie the mechanisms of viral induction of the disease [9, 10, 11, 12, 13, 14, 15]. The immunoglobulin repertoire of MS patients contains cross-reactive antibodies capable of simultaneously binding the human myelin basic protein and components of the Epstein–Barr virus [15, 16]

Regulatory B cells (Bregs), a new subpopulation of B cells, have recently become the object of increasing attention [17,18]. The fundamental interest in them lies in the need to understand the exact mechanism of suppression of the inflammatory response by B cells. There is no clear understanding at what stage of maturation a B cell acquires regulatory functions and how it is affected by BCR specificity. From a practical point of view, Bregs attract one’s attention as the cells directly involved in the development of autoimmune and lymphoproliferative pathologies. However, it is impossible to draw an unambiguous conclusion about the exact deviations that happen during autoimmune inflammation: there can be a change in the number of Breg cells, a disruption of their functions, or a combination of these two phenomena. Furthermore, there is limited information regarding the specificity of Bregs, although it has been shown that they require a B-cell receptor for proper functioning [19]. It is unclear whether the development of an autoimmune response is accompanied by disruptions in the maturation of Breg immunoglobulin genes and whether these regulatory cells can be autoreactive. It is not known at what stage of development the most significant changes in the Breg pool occur: in naïve, transitional, or mature B cells? Earlier, we found an increased number of transitional Bregs (tBregs) in the peripheral blood of MS patients [20]. Notably, the tBregs’ immunoglobulin heavy chains in MS patients carry fewer hypermutations compared to healthy donors.

In the present study, we have examined whether the structures of B-cell receptors from one of the most fully described subpopulations of tBregs, CD19+CD24highCD38high, differ in MS patients and healthy donors. The NGS analysis of BCR sequences (BCR-Seq) revealed that the distribution of a number of immunoglobulin germline genes in MS patients differs from that in healthy individuals. Moreover, during our analysis of the total pool of B cells and the tBreg subpopulation, both an excess and decrease in germline occurrence were observed in comparison with the normal range. It is important to note that this difference is more pronounced in patients with a more severe disease course (highly active MS (HAMS) [21]) compared to those with benign MS (BMS) [22].

## EXPERIMENTAL

**MS patients and healthy donors **Peripheral blood was sampled from nine patients with MS and six healthy donors (Table 1) at the Sixth Neurological Department of the Research Center of Neurology (Moscow, Russia). The patients with MS were aged 23–61 years (mean, 40.0 ± 9.1). Disease severity according to the EDSS scale ranged from 1.5 to 8.5. EDSS scores from 0 to 10 were calculated using the Kurtzke Expanded Disability Status Scale (EDSS) [23]. Five patients with HAMS and four patients with BMS were selected for the study. Data on the disease course, as well as treatment duration and history, were collected (Table 1). The study was approved by the local ethics committee of the Neurology Research Center and was conducted in full compliance with the WMA Declaration of Helsinki, ICH GCP, and relevant local legislation. All patients provided written informed consent after discussion of the study protocol.

**Table 1:** List of MS patients and healthy donors participating in the study

No.	MS phenotype1	Age, years	Sex	EDSS^2^	Treatment^3^	Disease duration, years
MS1	BMS	56	female	2.5	No treatment	11
MS2	BMS	61	female	3	No treatment	26
MS3	BMS	43	female	1.5	No treatment	12
MS4	BMS	36	male	2.5	No treatment	14
MS5	HAMS	33	male	6	IFNβ1b (2006–2011; 2014–2017).	12
MS6	HAMS	23	male	5	No treatment	3
MS7	HAMS	37	female	5	IFNβ1b (2014–2016). GA (2016–2017).	5
MS8	HAMS	29	female	8	GA (2012–2014). IVIG (2014). IFNβ1b (2015–2016).	12
MS9	HAMS	39	female	8.5	No treatment	8
HD1	Healthy	24	female	–	–	–
HD2	Healthy	40	female	–	–	–
HD3	Healthy	36	male	–	–	–
HD4	Healthy	27	female	–	–	–
HD5	Healthy	42	female	–	–	–
HD6	Healthy	25	female	–	–	–

^1^BMS – patient with benign MS; HAMS – highly active MS; HD – healthy donor.

^2^EDSS – the Expanded Disability Status Scale.

^3^IFNβ1b – interferon-β-1b; GA – glatiramer acetate; IVIG – intravenous immunoglobulin.

**Isolation of B cells from peripheral blood **Mononuclear cells from the peripheral blood of MS patients and healthy donors were obtained by sedimentation enrichment using Ficoll density gradient centrifugation. The residual erythrocyte fraction was removed using a ACK lysing buffer. The resulting mononuclear cells were filtered through a 40-mm nylon filter and stained with fluorescent antibodies: α-CD19-PE-Cy7, α-CD24-PE, α-CD38-APC, α-CD45-APC-Cy7 (Bio-legend, USA), and SYTOX Green dead cell stain (ThermoFisher Scientific) for 60 min at +4°C in the dark. The populations of tBregs (CD19+CD24highCD38high) and total B cells (CD19+) were collected directly into microcentrifuge tubes containing a Qiazol lysis buffer (Qiagen, Germany). Cell sorting was performed using a BD FACSAria III flow cytometer.

**Library preparation for immunoglobulin sequencing (RT-PCR) **RNA isolation was performed using an RNeasy Mini Kit (Qiagen, Germany) according to the manufacturer’s protocol. Reverse transcription (RT) was carried out using an MMLV RT kit with oligo(dT) and random primers according to the manufacturer’s in structions (Evrogen, Russia). Oligonucleotides for the amplification of variable fragments of human immunoglobulins VH and VL contained 15 forward primers for VH and four reverse primers for the human heavy chain J fragment, 13 Vκ forward primers and two Jκ reverse primers for the kappa light chain, and 16 Vλ forward primers and three reverse primers Jλ for the lambda light chain [24]. Fifteen VH forward primers were used individually in each sample in a 50 μL reaction mixture with an equimolar mixture of four JH reverse primers. Thirteen Vκ primers and sixteen Vλ primers were used individually to amplify the VL genes with an appropriate mixture of two Vκ reverse primers or three Vλ reverse primers in 50 μL of the reaction mixture for each sample. cDNA (0.02 μg) was used as a template in PCR performed with the Hot Start Taq Master Mix kit (Evrogen, Russia). The PCR conditions were as follows: one step (94°C – 3 min); one cycle (94°C – 25 s, 62°C – 25 s, 72°C – 25 s); two cycles (94°C – 25 s, 60°C – 25 s, 72°C – 25 s); two cycles (94°C – 25 s, 58°C – 25 s, 72°C – 25 s); three cycles (94°C – 25 s, 56°C – 25 s, 72°C – 25 s); three cycles (94°C – 25 s, 54°C – 25 s, 72°C – 25 s); 30 cycles (94°C – 25 s, 52°C – 25 s, 72°C – 25 s); and final elongation (72°C – 4 min). PCR mixtures of 15 VH gene samples, 13 Vκ gene samples, and 16 Vλ gene samples were individually pooled and concentrated to 50–80 μL using an Amicon 30 kDa centrifugal filter unit (Merck, Millipore). The PCR products (~ 400 bp) VH, Vκ, and Vλ were loaded onto 1.5% agarose gel and purified using an agarose gel DNA purification kit (Monarch, NEB).

**Next-generation sequencing of VH, Vκ, and Vλ variable immunoglobulin fragments**

One μg of purified VH, Vκ, and Vλ PCR product was ligated to NEBNext Multiplex Oligos adapters using the NEBNext Ultra DNA Library Preparation Kit for Illumina (NEB). Libraries were sequenced on a MiSeq system using a 2 × 300 bp sequencing kit (Illumina) at the Genomics Core Facility SB RAS (Institute of Chemical Biology and Fundamental Medicine SB RAS, Novosibirsk, Russia).

**Analysis of the NGS data**

The analysis was carried out using the MiXCR software [25] in two stages. Initially, raw sequencing data were processed using the default MiXCR algorithm (align, assemble, export) employing the IMGT library as a germline gene reference. The generated reads successfully aligned with the germline genes and containing the complete immunoglobulin target sequence (CDR1 + FR2 + CDR2 + FR3 + CDR3) were then subjected to resampling to normalize different numbers of reads. When analyzing the occurrence frequency of germline genes, mutations in the variable fragments VH, Vκ, and Vλ were not taken into account.

**Statistical analysis**

The statistical analysis was performed using the Prism 6 software utilizing the Mann–Whitney test and paired Student’s t-test.

## RESULTS AND DISCUSSION

Recently, there has been a growing number of studies demonstrating the importance of B cells in the regulation of autoimmune diseases, including MS [26, 27]. However, the Breg subpopulations in MS patients still have not been fully characterized. To date, very little data have been published on the specificity and structure of their B-cell receptors. For a deeper understanding of the nature of Bregs development and characterization of their maturation, we analyzed the CD19+CD24highCD38high subpopulation, one of the most convincingly-confirmed phenotypic portraits of tBregs, which are at an intermediate stage of development between immature bone marrow cells and fully mature naïve B cells in peripheral blood and secondary lymphoid tissues [28, 29]. Peripheral blood samples were obtained from nine patients with MS and six healthy donors (Table 1). Mononuclear cells were stained with antibodies against the CD19, CD24, and CD38 surface markers. The total pool of CD19+ B cells and tBreg CD19+CD24highCD38high were separately obtained by cell sorting for subsequent RNA isolation and sequence analysis of B-cell receptors. For this purpose, the sequences of the variable fragments of the heavy (VH) and light (VL) immunoglobulin chains of each patient were amplified from cDNA synthesized from the isolated RNA, and, then, NGS of the VH, Vκ, and Vλ genes was performed. For a fair analysis, sequencing of the immunoglobulin repertoire of the total B-cell pool and the subset of transitional Bregs was performed with a read depth of at least five functional reads per sorted cell. After all the stages of bioinformatic filtering, we obtained an average of 83,100 functional sequences for the heavy chain; 37,591 sequences for the kappa chain; and 34,565 sequences for the lambda chain of the total pool of CD19+ cells and tBreg subpopulation with the CD19+CD24highCD38high phenotype. The sequences are available in the ArrayExpress repository (https://www.ebi.ac.uk/arrayexpress/experiments/E-MTAB-10859).

**Fig. 1:**
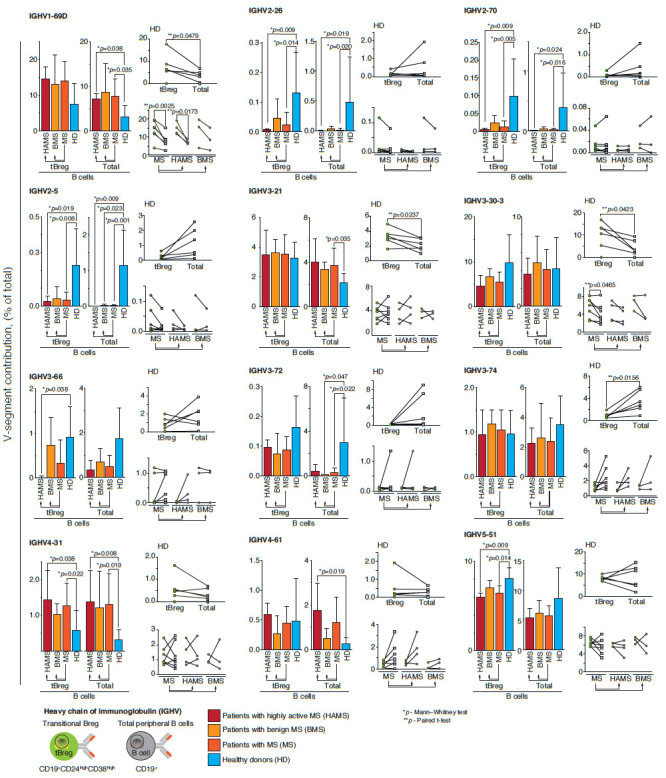
Differential usage of germline Ig VH-gene segments in MS patients and healthy donors. The frequency of 49 functional VH genes in patients with MS, separately for BMS and HAMS, was analyzed. The frequency of VH germline genes in healthy donors (HD) was analyzed as a control. The distribution of the germline gene repertoire was compared between the total pool of peripheral blood B cells (CD19+) and tBregs with the CD19+CD24highCD38high phenotype. Histograms show the comparison of patients with different types of MS courses to healthy donors, where the average value (mean ± SD) of the proportion of IgG sequences related to the indicated germline is provided for each group. The individual values of the proportion of immunoglobulin germline genes for each patient are compared for the total pool of B cells (total, gray dots) and the subpopulation of transient regulatory B cells (tBregs, green dots) and shown to the right of the histogram. The data are provided only for the germline genes for which a statistically significant difference was shown in at least one analyzed parameter (comparison of different types of MS courses against healthy donors was performed using the Mann–Whitney test; comparison of the total pool of B cells with the tBreg subpopulation was performed using the paired t-test; only statistically significant *p*-values are shown)

To analyze the distribution of the VH, Vk, and Vλ germlines, we used primers capable of amplifying almost all possible variants of the VHDJH, VKJK, and VλJλ. functional fragments. When comparing the distribution of IgVH genes in patients with MS and healthy donors, all seven functional families of VH were amplified. The IGHV3 germline genes were most abundant in MS patients and healthy donors in both the total B-cell pool and the tBreg subpopulation. The IGHV2-26, IGHV2-5, IGHV2-70 germline immunoglobulin sequences, which are present in small amounts in almost every healthy individual, disappear during the development of MS (Fig. 1). In patients with a more severe disease (HAMS), the variation in the repertoire of tBreg immunoglobulin genes increases as compared to healthy donors. The IGHV3-66 germline occurs in healthy donors and patients with BMS at a comparable level, but this gene almost completely disappears in HAMS patients. One of the major germlines, IGHV5-51, is observed in all the analyzed donors, but its frequency decreases significantly in MS patients. Contrariwise, the only IGHV4-31 gene is more frequent in MS patients both in the total pool of B cells and in the tBreg subpopulation. This correlates with the previously published data on increased levels of the IGHV4 family in the B-cell repertoire of the peripheral blood and cerebrospinal fluid of MS patients [30, 31].

**Fig. 2:**
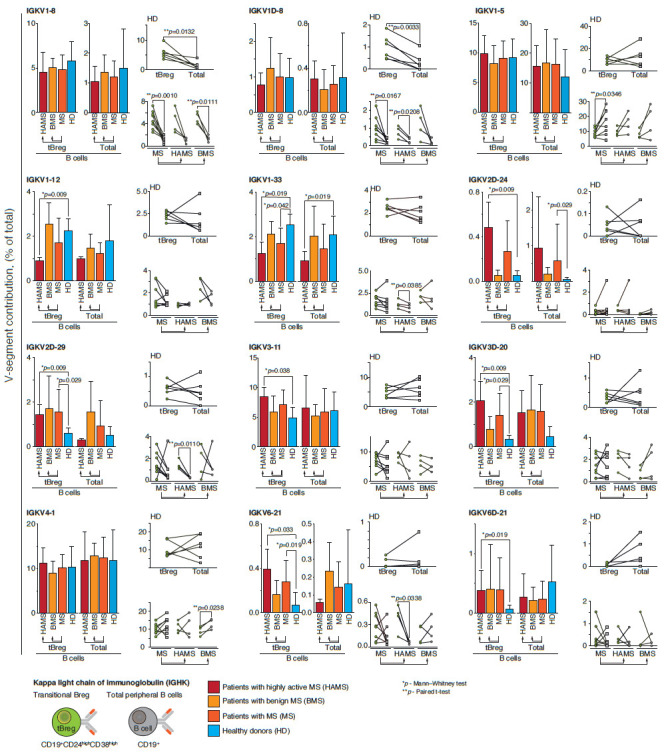
Differential usage of germline Ig Vk-gene segments in MS patients and healthy donors. Frequency of 41 functional Vk genes in MS patients, separately for BMS and HAMS patients, was analyzed. The frequency of Vk germline genes in healthy donors (HD) was analyzed as a control. The distribution of the germline gene repertoire was compared between the total pool of peripheral blood B cells (CD19+) and tBregs with the CD19+CD24highCD38high phenotype. Histograms show the comparison of patients with different types of MS course and healthy donors, where the average value (mean ± SD) of the proportion of IgG sequences related to the indicated germline is provided for each group of patients. Individual values of the proportion of immunoglobulin germline genes for each patient are compared for the total pool of B cells (total, gray dots) and the subpopulation of transient regulatory B cells (tBregs, green dots) and shown to the right of the histogram. The data are provided only for germline genes, for which a statistically significant difference was shown in at least one analyzed parameter (comparison of patients with different types of MS course against healthy donors was performed using the Mann–Whitney test; comparison of the total pool of B cells with the subpopulation of tBregs was performed using the paired t-test; only statistically significant *p*-values are shown)

The repertoire of germline genes encoding light chains of Breg immunoglobulins also differs between MS patients and healthy donors. The portion of IGKV1-12 germline, which is normally found in approximately 2% of immunoglobulin sequences, decreases below 1% in the case of HAMS patients (Fig. 2). In BMS patients with a milder disease course, the frequency of the IGKV1-12 germline does not differ from that in healthy donors. The IGKV1-33 germline is less common not only in HAMS patients, but also in the entire group of MS patients, which includes patients with both disease courses. On the contrary, IGKV2D-24, IGKV3-11, and IGKV6D-21 germline genes are significantly more common in HAMS patients than in healthy donors. The IGKV2D-29, IGKV3D-20, and IGKV6-21 genes are more prevalent both in HAMS patients and in the MS group in general. For the kappa light chain, the distribution of germline genes in the tBreg population does not significantly differ between BMS patients and healthy donors.

**Fig. 3:**
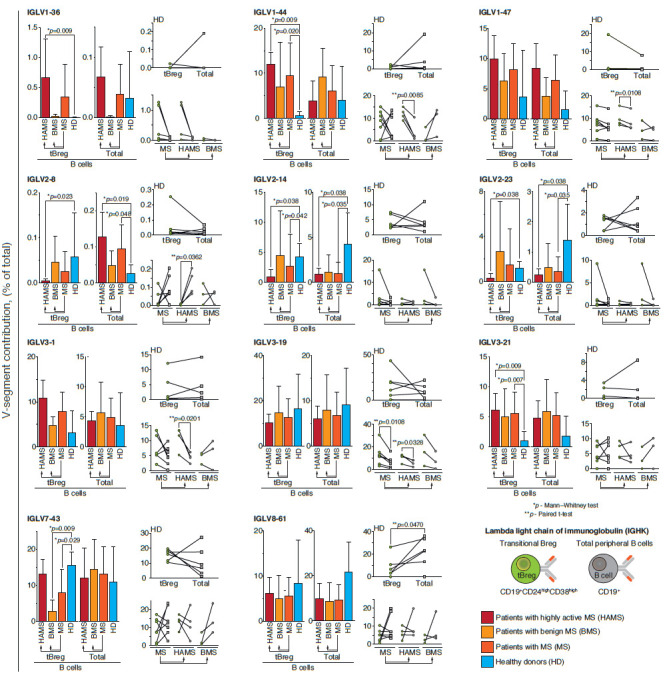
Differential usage of germline Ig Vλ-gene segments in MS patients and healthy donors. Frequency of 26 functional V**λ **genes in MS patients, separately for BMS and HAMS patients, was analyzed. The frequency of V**λ **germline genes in healthy donors (HD) was analyzed as a control. The distribution of the germline gene repertoire was compared between the total pool of peripheral blood B cells (CD19+) and tBregs with the CD19+CD24highCD38high phenotype. Histograms show a comparison of patients with different types of MS courses and healthy donors, where the average value (mean ± SD) of the proportion of IgG sequences related to the indicated germline is provided for each group. Individual values of the proportion of immunoglobulin germline genes for each patient are compared for the total pool of B cells (total, gray dots) and the subpopulation of transient regulatory B cells (tBregs, green dots) and shown to the right of the histogram. The data are provided only for the germline genes for which a statistically significant difference was shown in at least one analyzed parameter (comparison of patients with different types of MS courses against healthy donors was performed using the Mann–Whitney test; comparison of the total pool of B cells with the subpopulation of tBregs was performed using the paired t-test; only statistically significant *p*-values are shown)

Differences in the distribution of immunoglobulin germline genes in tBregs during MS development are also observed in the case of the lambda light chain isotype (Fig. 3). The IGLV1-36 germline is almost never observed in healthy donors and BMS patients, but its frequency significantly rises to 0.5% in HAMS patients. The frequency of IGLV1-44 and IGLV3-21 germline genes is increased in patients with any type of MS course; however, a statistically significant difference is observed only between HAMS patients and healthy donors. The distribution of IGLV2-8, IGLV2-14, and IGLV2-23 germline genes does not differ between BMS patients and healthy donors, but their frequency significantly decreases with the development of HAMS. Interestingly, representation of the IGLV7-43 germline gene, conversely, is approximately the same in HAMS patients and healthy donors, but significantly decreases in BMS patients.

## CONCLUSIONS

Immunological studies carried out in the 21^st^ century have confirmed the crucial role of Bregs in maintaining immunotolerance, as well as controlling and reducing the inflammatory response. There are still many questions regarding the exact mechanism of its regulation, but it is obvious that a violation of the number and function of Breg cells leads to the development of various immunological pathologies, among which MS is particularly prominent. A detailed elucidation of inflammatory regulation by B cells will allow us not only to determine the etiology of autoimmune pathologies, but also may contribute to the development of Breg-based therapy in the near future. Immunoglobulins play an important role in the immune response by being exposed as antigen-specific receptors on the B-cell surface, as well as secreted antibodies. The recent progress achieved in the NGS analysis makes it possible to identify immunoglobulin repertoires with an unprecedented high level of detailing [32]. Therefore, it is extremely important to study the structure and functions of immunoglobulins, their specificity, and epigenetic status to understand the fundamental principles of MS onset and progression. Over the recent years, more and more patterns and stereotyped antibody responses have been discovered, when different individuals produce immunoglobulins recognizing certain antigenic epitopes using the same IgV genes [32, 33, 34]. In other words, certain immunoglobulin germlines exhibit tropism to certain antigens. Accordingly, variations in the usage of some immunoglobulin germline genes can be associated with a different antibody’s ability to generate an effective immune response, which may manifest itself as predisposition to various diseases, including autoimmune ones. It is likely that the differences in the germline gene frequency in each individual can be the result of an antiviral or autoimmune response. Before the onset of antigen-dependent B cell differentiation mediated by somatic hypermutation of immunoglobulin sequences, the diversity of immature B cells, including tBregs, is almost entirely determined by the configuration of the body’s germline genes (V(D)J recombination). Therefore, a detailed study of the immunoglobulin repertoire of immature B cells in patients with various autoimmune diseases, including MS, will help determine which rearrangements in the immunoglobulin germline genes can lead to functional disorders of the immune system.

The present study revealed that the distribution of immunoglobulin germline genes in the tBreg population in MS patients differs from that in a healthy person. Particularly significant differences are observed for the IGLV1-44 and IGHV2-5 germlines. The IGLV1-44 lambda chain germline is almost absent in the tBreg subpopulation of a healthy person but is found in MS patients. The IGHV4-31 germline is more frequent during MS development, both in the total pool of B cells and in the tBreg subpopulation. The opposite situation is observed for the IGHV2-26, IGHV2-5, IGHV2-70 germline genes of the heavy chain: these germlines, although being identified at small amounts in almost every healthy individual, disappear in MS patients. Moreover, in the case of a severe form of the disease, the difference from the normal value becomes larger. We have also previously found more significant differences in the number of peripheral tBregs and their maturation level in HAMS patients compared with BMS patients and healthy donors [20]. Therefore, this study has shown that a more significant variation in the tBreg CD19+CD38highCD24high subpopulation repertoire is associated with a more aggressive MS course. In general, a similar situation is observed for all the germlines but IGLV1-44: if the immunoglobulin germline frequency in the total pool of circulating B cells changes during MS development, a similar picture is also true for tBregs. Therefore, during the development of the autoimmune MS pathology, disruptions in the distribution of the immunoglobulin germline genes can be genetically predetermined and occur already at an early stage of B-cell maturation. To confirm this hypothesis, the size of the analyzed patients’ cohorts needs to be increased and the differences in the structure and specificity of the B-cell receptors of other Breg subpopulations need to be studied.

## Abbreviations

MS: multiple sclerosis
CNS: central nervous system
HAMS: highly active multiple sclerosis
BMS: benign multiple sclerosis
Breg: regulatory B cell
tBreg: transitional regulatory B cell
